# Inhibition of Aldose Reductase by Novel Phytocompounds: A Heuristic Approach to Treating Diabetic Retinopathy

**DOI:** 10.1155/2022/9624118

**Published:** 2022-03-21

**Authors:** Angeline Julius, Remya Rajan Renuka, Waheeta Hopper, P. Babu Raghu, Sharmila Rajendran, Senthilkumari Srinivasan, Kuppamuthu Dharmalingam, Amer M. Alanazi, Selvaraj Arokiyaraj, S. Prasath

**Affiliations:** ^1^Centre for Materials Engineering and Regenerative Medicine, Bharath Institute of Higher Education and Research, Chennai 600126, India; ^2^Department of Biotechnology, School of Bioengineering, Faculty of Engineering and Technology, SRM Institute of Science and Technology, Chennai 603203, India; ^3^Department of Zoology, Madras Christian College, Affiliated to University of Madras, Chennai 600059, India; ^4^Department of Ocular Pharmacology, Aravind Medical Research Foundation, Dr. G. Venkataswamy Eye Research Institute, Madurai 625020, India; ^5^Department of Proteomics, Aravind Medical Research Foundation, Dr. G. Venkataswamy Eye Research Institute, Madurai 625020, India; ^6^Pharmaceutical Chemistry Department, College of Pharmacy, King Saud University, Riyadh 11451, Saudi Arabia; ^7^Department of Food Science and Biotechnology, Sejong University, Seoul, Republic of Korea; ^8^Department of Mechanical Engineering, College of Engineering and Technology, Mizan Tepi University, Tepi, Ethiopia

## Abstract

Aldose reductase (ALR2) activation in the polyol pathway has been implicated as the primary mechanism for the progression of diabetic retinopathy. Most of the aldose reductase inhibitors (ARIs), used for the treatment of diabetic complications, were withdrawn due to ineffective treatment and adverse side effects caused by nonspecificity. Epalrestat, a carboxylic acid inhibitor, is the only ARI used for the treatment of diabetic neuropathy, though associated with minor side effects to 8% of the treated population. Our study exploited the interactions of Epalrestat-ALR2 crystal structure for the identification of specific phytocompounds that could inhibit human lens ALR2. 3D structures of plant compounds possessing antidiabetic property were retrieved from PubChem database for inhibition analysis, against human lens ALR2. Among the shortlisted compounds, Agnuside and Eupalitin-3-O-galactoside inhibited lens ALR2 with IC50 values of 22.4 nM and 27.3 nM, respectively, compared to the drug Epalrestat (98 nM), indicating high potency of these compounds as ALR2 inhibitors. IC50 concentration of the identified ARIs was validated in vitro using ARPE-19 cells. The in silico and in vitro approaches employed to identify and validate specific and potent ALR2 inhibitors resulted in the identification of phytocompounds with potency equal to or better than the ALR2 inhibiting drug, Epalrestat.

## 1. Introduction

Diabetes is a metabolic disorder characterized by hyperglycemia that results from defective insulin secretion, insulin action, or both [1]. Globally, in 2005, 151 million patients were reported to have type 2 diabetes, of which 50% were middle-aged. A fourfold increase in diabetes patients was reported between 2006 and 2014 and is expected to double by 2025 [2]. With response to prolonged hyperglycemia, diabetes-associated complications affect the vasculature and organs including the eyes, kidneys, and heart [3].

Complications of diabetes are categorized as microvascular complications that include nephropathy, retinopathy, and neuropathy and macrovascular complications including cardiovascular disease, heart attacks, and stroke that occur due to severe pathogenesis in both type 1 and type 2 diabetes [4]. More than one-third of patients with diabetes have been reported to have at least one microvascular complication [5]. Polyol pathway is the first pathway identified to cause diabetic complications followed by the involvement of advanced glycation end products (AGEs) and hyperglycemia-induced isoforms of protein kinase C (PKC) [6]. Aldose reductase (ALR2), the rate limiting enzyme of the polyol pathway, catalyzes nicotinamide adenosine dinucleotide phosphate (NADPH)-dependent reduction of glucose to sorbitol, which is then converted to fructose. ALR2 gets activated during hyperglycemia causing an increase in intercellular sorbitol accumulation, AGEs production [7], and vasoconstriction factors that cause hypoxia and activate the pathways of neovascularization [8]. Furthermore, ALR2 increases the expression of inflammatory cytokines and vascular endothelial growth factor (VEGF) through the activation of nuclear factor-*κ*appaB (NF-*κ*B) that invokes the phosphatidylinositol-3-kinase/Akt signaling (PI3-Akt) pathway for cell survival, proliferation, and migration, and facilitates neovascularization [9]. Excessive consumption of NADPH due to activation of ALR2 causes an increase in redox stress. Since NADPH is required to regenerate reduced glutathione (GSH), which is the scavenger of reactive oxygen species (ROS), its depletion could cause intracellular oxidative stress. Excessive production of ROS causes tissue damage in different organs [10]. ALR2 involvement in the pathology during hyperglycemia makes its role evident in causing complications.

Among the diabetic complications, diabetic retinopathy (DR) is the most common and severe complication and the leading cause of blindness in developed countries. Localization of ALR2 in the retina and its activity cause an increase in vascular permeability, formation of acellular capillaries, cell loss, and capillary basement membrane thickening at early stages followed by neovascularization associated proliferative DR [11]. An increase in ALR2 activation in the retinal cells with response to hyperglycemia has been demonstrated. An Increase in ALR2 activity causes damage to the retinal endothelial cells [12], pericytes [13], retinal pigment epithelial cells, and Müller cells [14]. Hyperglycemia-related ALR2 activation causes cell damage with reduced viability of the retinal cells that could be reversed or prevented using aldose reductase inhibitors (ARIs) [15]. These findings provide enough evidence for the potent use of ALR2 for the treatment of DR.

Delay in treatment for DR accounts for worsening and rapid progression; hence, early detection and follow-up are necessary since DR shows few retinal abnormalities before progressing to the irreversible stage [16]. Novel pharmacological interventions for the reduction of oxidative stress and control of the progression of DR are required for effective treatment [17]. Laser therapy, intravitreal steroid injections, anti-VEGF therapy, photocoagulation, and vitrectomy are used in combination for the treatment of DR. However, vision loss occurs despite the treatment [18]. Though the current techniques try to prevent vision loss, poor response and side effects from the drugs used have been noted in many of the cases [19]. Laser photocoagulation therapy is commonly used to treat DR and has been also reported to cause retinal damage [20]. Thus, novel treatment strategies, which could control DR, would be a heuristic approach.

The most critical step in controlling DR is controlling the factors associated with the progression of DR. Since ALR2 inhibition could potentially control the factors associated, efficient inhibition of ALR2 by potent ARIs is necessary [21]. Initially, two classes of ARIs belonging to the class of spirosuccinimide and carboxylic acids were broadly in use. Later, orally active pyridazinones, which showed higher selectivity for ALR2 than other AKRs, were identified [22]. Early detection and control of the blood glucose in diabetes would reduce the risk of developing DR [23].

Since current antidiabetic therapies are not cost-effective and include severe side effects that worsen the condition, phytocompounds are considered safe, effective, and cost-effective in the treatment of DR [24]. Herbal medicines that offer antidiabetic properties are used in adjuvant therapy in addition to other antidiabetic treatments for effective management and control of DR. In this regard, clinical and experimental studies on herbal drug interactions have to be exploited further, and this research could be an eye-opener for the search of multitarget drugs [25].

Though a number of ALR2 inhibitors have been developed and tested, none of them were clinically successful because of the limited efficacy and safety issues [26]. Natural product research has recently gained interest due to the failure of alternative drug discovery in delivering potent lead compounds for the treatment of a variety of diseases [27]. About 40% of the medicines marketed are natural products or their semisynthetic derivatives [28]. Phytocompounds have widely contributed to primary healthcare and are used as a source for a novel drug discovery process to treat various ailments [29–33]. Among the phytochemicals identified so far, quercetin, kaempferol, and ellagic acid are the most promising natural ALR2 inhibitors [34].

Epalrestat is the only FDA-approved ARI to date used for the treatment of diabetic neuropathy. Employing in silico and *in vitro* advancements to identify potent phytocompounds would benefit future research. Identification of specific inhibitors that potentially bind to ALR2, among other aldo-ketoreductases (AKRs) such as aldehyde reductase (ALR1) and ALR2–like protein (AKR1B10) that show structural similarities to ALR2, would control the side effects caused due to nonspecific binding that has led to the withdrawal of most of the inhibitors. This work exploits the information from the crystal structure of ALR2 complexed with the drug Epalrestat to identify specific inhibitors of ALR2 with equivalent potency to Epalrestat. Human lens ALR2 inhibition by the phytocompounds was further validated using ARPE-19 cells.

## 2. Materials and Methods

All in silico analysis including Glide docking, induced fit docking (IFD), and molecular dynamics (MD) were performed using Schrödinger LLC Maestro version 10.2 [35].

### 2.1. Preparation of Phytocompounds and ALR2 Protein Structure for In Silico Analysis

Phytocompounds from plants reported with antidiabetic property were retrieved from Duke's Database of Natural Compounds.  Table 1 provides the names of the plants with antidiabetic property. The 3D structure-data files of the plant compounds were retrieved from PubChem database. The retrieved phytocompounds were prepared using LigPrep module of Maestro, and the ligand structures were geometrically minimized using OPLS_2005 force field to obtain low energy 3D structural variants of the ligands. The optimized ligands were subjected to Glide docking based computational screening for the identification of potential lead compounds.

### 2.2. Preparation of ALR2 Protein Structure

Crystal structure of ALR2 complexed with NADP+ and drug Epalrestat—ID: 4JIR, the only available crystal structure of ALR2 complexed with Epalrestat—with 2.0 Å resolution was retrieved from Protein Data Bank (PDB). This protein structure was prepared using Protein Preparation Wizard protocol of Maestro.

### 2.3. Grid Generation and Glide Docking Protocol

For the protein, the receptor grid, which acts as a three-dimensional boundary, was generated for the binding ligand, depicting the active site region of the protein. The ligand binding sites in the protein were covered by the large grid with a midpoint diameter of 10 Å. Glide docking was performed for the phytocompounds to determine the ligand binding free energy.

### 2.4. Flexible Docking Analysis Using IFD

IFD was applied to determine the ALR2 protein and the phytocompounds binding interactions using Prime and Glide modules. The side chains of the amino acids of ALR2 were optimized using Prime refinement. Docking calculations were performed in XP mode, and the XP descriptors were noted.

### 2.5. Specificity Analysis of the Phytocompounds Using IFD Generated Protein-Ligand Interactions

Crystal structure of AKR1B10 complexed with NADP+ and Epalrestat (PDB ID : 4JIH) with a resolution of 2.3 Å and crystal structure of ALR1 holoenzyme in complex with the ALR2 inhibitor Fidarestat (3H4G) with a resolution of 1.85 Å were retrieved from the PDB. The phytocompounds shortlisted using IFD protocol were subjected to specificity study, to identify compounds that could specifically inhibit ALR2, compared to AKR1B10 or ALR1. The crystal structure files, 4JIH and 3H4G, corresponding to AKR1B10 and ALR1 protein were prepared using protein preparation wizard.

### 2.6. Analysis of the Stability of Protein-Ligand Complex Using 100 nm MD Simulation

Desmond 3.2 program was employed to confirm the stability of the protein-ligand complex after extensive 100 ns MD simulation within restrained conditions. MD simulations were performed with OPLS2005 molecular mechanics force field. The backbone root mean square fluctuations (RMSF) and root mean square deviations (RMSD) of the complexes were recorded for each trajectory throughout the simulation time. The RMSD and the RMSF of the backbone atoms were used to determine the change in confirmation of the protein and the average atomic mobility during a simulation event, respectively.

### 2.7. Cytotoxicity Assay

The cell toxicity of the shortlisted ARIs was assessed using MTT assay kit [36] (*in vitro* toxicological assay kit, Sigma, USA). ARPE-19 cells were grown to confluence in DMEM/F12 medium with 10% FBS, harvested by trypsinization, and plated at 1 × 10^4^ cells per well in a 96-well plate. The medium was replaced with serum-free medium without FBS for 3 hr before the drug treatment. Following serum starvation, ARPE-19 cells were treated with different concentrations of the identified compounds. Epalrestat was used as the positive control for inhibition. The cells were treated with the lead compounds for 24 hr to determine the cytotoxicity of the compounds. After incubation, the cells were treated with 20 *μ*l of 5 mg/ml MTT and incubated for 3 hr at 37°C to determine the cell toxicity. The viable cells converted MTT into formazan crystals which were dissolved using solubilization solution (200 *μ*l) and measured spectrophotometrically at 570 nm with subtraction of the reference wavelength, 650 nm, to eliminate the possibility of variations in the plate and the media present, using SpectraMax M3 multiplate reader (Molecular Devices, California, USA).

### 2.8. Isolation of ALR2 Enzyme from Human Lens

Hayman and Kinoshita [37] method of crude ALR2 enzyme preparation was followed. Five diabetic and nondiabetic human lenses, aged 58 to 65 years, were obtained from the Rotary Aravind International Eye Bank, Aravind Eye Hospital, Madurai. Crude ALR2 enzyme extract was prepared by homogenizing from 100 mg of diabetic and nondiabetic human lenses separately in 1 ml of 135 mM sodium potassium phosphate buffer of pH 7.0, containing phenylmethylsulfonyl fluoride (PMSF) and 10 mM 2-mercaptoethanol. The nondiabetic human lens was used as the control. The lens homogenate was centrifuged at 10,000 rpm for 30 minutes, and the resulting crude ALR2 enzyme preparation was used for analysis.

Specific activity of ALR2 present in the crude sample was calculated using the following formula:

(1) $$\begin{array}{c} \text{Acitivity}\frac{U}{ml}=\frac{\text{Change in OD of test}/\min\times \text{total volume of the assay}}{6\text{.}2\times \text{Volume of enzyme taken for analysis}}, \end{array}$$

where 6.2 is the micromolar extinction coefficient of NADPH at 340 nM.

(2) $$\begin{array}{c} \text{Specific activity}=\frac{\text{Activity}\left(U/ml\right)}{\text{Total protein}\left(mg/ml\right)}\text{.} \end{array}$$

### 2.9. ALR2 Inhibition in Human Lens Samples

The 300 *μ*l reaction mixture consisted of 100 mM sodium phosphate buffer adjusted to pH 7.0, 0.2 mM Li_2_SO_4_, 5 mM 2-mercaptoethanol, 0.15 mM NADPH, and 100 *μ*g crude enzyme preparation, and the substrate DL-glyceraldehyde was added to initiate the reaction. ALR2 activity was assayed spectrophotometrically by measuring the decrease in the absorption of NADPH at 340 nm per unit time at 37°C and pH 7.0.

The percentage inhibition of ALR2 was calculated using the following formula:

(3) $$\begin{array}{c} \%\text{ inhibition}=\frac{\left(\Delta \text{Absorbance of sample}/\min-\Delta \text{Absorbance blank}/\min\right)}{\left(\Delta \text{Absorbance of control}/\min-\Delta \text{Absorbance blank}/\min\right)}\times 100. \end{array}$$

The concentration of inhibitors inhibiting 50% of the enzyme activity (IC_50_) was calculated using GraphPad Prism 7 software from the least-squares regression line of the logarithmic concentrations plotted against the residual activity [38].

### 2.10. Isolation of ALR2 Crude Enzyme from ARPE-19 Cells

ARPE-19 cells, 2.5 × 104/well, were grown in 1 ml of Dulbecco's Modified Eagle Medium: Nutrient Mixture F12 (DMEM/F12) with 10% fetal bovine serum (FBS) under normoxia to near confluency. The cells were shifted to DMEM-low glucose with 10% FBS, 12 hr before the experiment. The cells were washed with PBS and incubated in FBS-free medium and 5 mM/25 mM (normoglycemic condition/hyperglycemic condition) glucose, for 24 hr, with or without the drug. Following the drug treatment, the cells were washed with ice cold PBS, trypsinized, and counted. Cells were resuspended in ALR assay buffer, homogenized for 30 seconds, and centrifuged at 10000 rpm for 30 minutes. The cell lysate was collected for analysis.

### 2.11. Inhibition of ALR2 in ARPE-19 Cells

ALR2 inhibition protocol by Hayman and Kinoshita was followed [34]. The IC_50_ value of the compounds inhibiting human lens ALR2 was checked for the inhibition of ALR2 in ARPE-19 cells.

## 3. Results and Discussion

### 3.1. Determination of ALR2 Protein-Ligand Binding Free Energy Using ALR2 Receptor Grid-Based Glide Docking

Glide docking of Epalrestat was performed on the binding site of ALR2 to determine the protein-ligand binding free energy, to shortlist compounds having similar binding affinity to that of the Epalrestat. Screening of the phytocompounds from plants reported with antidiabetic activity, against the binding site grid of ALR2, resulted in the selection of 16 compounds based on a lower Glide score than that of Epalrestat (−7.641 Glide score), indicating high affinity of the compounds for ALR2 protein. Literature survey indicated that all these compounds except Eupalitin-3-O-galactoside, Picroside II, Agnuside, and 7-O-Methylwogonin were inhibitors of ALR2 protein. The occurrence of already reported ARIs among the shortlisted compounds validates the protocol of Glide docking, which accurately docks and scores compounds according to the binding affinities of the compounds under screening. The shortlisted natural compounds had nearly a twofold increase in affinity for ALR2 compared to Epalrestat. Development of novel ARIs with high efficacy has been most disappointing due to discrepant doses that are given for the treatment [39]. ARIs withdrawn due to the lack of efficiency were successful when given in high dose levels but were toxic to the liver. Discovery of novel drugs capable of inhibiting ALR2 with higher efficacy and safety than the withdrawn ARIs would be a heuristic approach to treating DR. The compounds shortlisted with higher affinity for ALR2 protein using Glide docking approach are listed in Table 2.

### 3.2. Analysis of Specificity Using Flexible Docking of the Identified Ligands against ALR2, ALR1, and AKR1B10 Protein Structures

The shortlisted compounds had low Glide scores with ALR2 protein compared to those of AKR1B10 and ALR1, indicating high affinity of the compounds for ALR2. The IFD Glide score of Epalrestat was −9.147, −7.242, and −8.516 with proteins ALR2 AKR1B10, and ALR1, respectively, inferring increased affinity for ALR2, but with not much difference. The IFD scores of compounds identified as inhibitors of ALR2 with the AKRs, ALR2, ALR1, and AKR1B10 are listed in Table 3. Among the identified compounds for ALR2 inhibition, Eupalitin-3-O-galactoside had the lowest IFD Glide score (−16.266 kcal/mol), followed by Agnuside (-15.302 kcal/mol), Picroside II (−13.000 kcal/mol), and 7-O-Methylwogonin (11.968 kcal/mol).

The binding mode differences of the identified ALR2 inhibitors on the other structurally similar AKR proteins, AKR1B10 and ALR1, could be used to identify specific inhibitors of ALR2.  Table 4 lists the amino acid interactions established by the identified ALR2 inhibitors on the AKRs.

The identified ligands had specific interactions with ALR2. Though the ligands had interactions with the active site residues of ALR1, which are homologous to the residues of ALR2, they had less affinity for ALR1 due to the significant difference in the active site region of ALR1, by the insertion of eight-residue segment (Asp306, Gly307, Lys308, Arg309, Val310, Pro311, Arg312, and Asp313) in the C-terminal region and the substitution of Leu300 and Thr113 by Pro301 and Tyr115, respectively [40]. Epalrestat had side chain hydrogen bond interactions with the active site residues Tyr48, Hie110, and Trp111. The identified compounds for ALR2 inhibition showed interactions with the specificity pocket residues in addition to the hydrophobic active site residues. Eupalitin-3-O-galactoside established *π*-*π* interaction with Phe122; side chain hydrogen bond interactions with Tyr48 and Hie110; and backbone hydrogen bond interactions with Val297, Ala299, and Ser302. Agnuside had backbone hydrogen bond interactions with Tyr48, Trp219, and Ser302. Picroside II established side chain hydrogen bond interactions with Trp20 and backbone hydrogen bond interactions with Tyr48, polar Lys21, Val47, and Ala299 residues. The 7-O-Methylwogonin had two backbone hydrogen bond interactions and one *π*-*π* interaction with Leu300, Leu301, and Hie110, respectively. Inhibitor binding to the anionic binding site near the NADP^+^ binding cleft of the active site region opens up the specificity pocket that helps in the binding of the inhibitors that are more effective and specific to ALR2 than ALR1 [41]. The identified ALR2 inhibitors had interactions with the specificity pocket residues of the ALR2 protein in addition to the anionic active site residues indicating high specificity. The failure of most of the ARIs used earlier was primarily due to two main reasons, ineffective treatment and adverse effects. ALR2 inhibitors, Fidarestat and Ranirestat, were ineffective in treating diabetic neuropathy and still are currently in phase III clinical trials. Other ARIs, Ponalrestat, Zopolrestat, Zenarestat, Tolrestat, and Alrestatin, were withdrawn due their nonspecificity against ALR2. Sorbinil, a potent ARI, was withdrawn after clinical trials due to lack of specificity resulting from a similar pattern of interaction with ALR2 enzyme and ALR1 with similar IC_50_ values. The active site region of ALR1 differs significantly from the active site of other AKR family members by the insertion of eight-residue segment (Asp306, Gly307, Lys308, Arg309, Val310, Pro311, Arg312, and Asp313) in the C-terminal region. Interaction of Leu300 of the ALR2 with the inhibitor is the key determinant of specificity of ALR2 over ALR1. The specificity of the compounds towards ALR2 was checked compared to other AKRs to rule out the nonspecificity issue. Epalrestat, the only available inhibitor of ALR2, did not show interactions with the specificity pocket residues of ALR2, which may be the reason for the adverse side effects caused by the prolonged treatment of Epalrestat [42]. The phytocompounds Eupalitin-3-O-galactoside, Picroside II, Agnuside, and 7-O-Methylwogonin were identified as potential ALR2 leads, based on their high binding affinity for ALR2.

### 3.3. Analysis of the Stability of the ALR2-Phytocompound Complexes

The ALR2-phytocompound complexes were subjected to MD simulations for 100 ns to determine the stability of the ligand within the complex. RMSD and RMSF were recorded for the simulation event, and the protein-ligand interactions were analyzed. The significant IFD interactions were checked for consistency throughout the simulation event.  Table 5 lists the average RMSD and RMSF of the simulated ALR2-phytocompound complexes. The complexes of ALR2 protein with identified compounds had less fluctuations and deviations compared to the ALR2-Epalrestat complex, indicating the stability of these complexes. Fluctuations were high from 217 to 223 residues, in each complex that corresponds to the cofactor binding loop region of the protein. The natural ligand in each simulated complex was well adapting in the binding site of the protein with less average RMSD and RMSF, compared to Epalrestat with an average RMSD and RMSF of 1.95 Å and 0.86 Å, respectively. Compared to other complexes, ALR2-Agnuside complex had lowest fluctuations and deviations indicating high stability of the complex.

The interactions established by Epalrestat in IFD, His110, and Tyr48 maintained 100% hydrogen bonding and 95% hydrogen bond interaction throughout the simulation event. The Trp111 hydrophobic interaction was not maintained consistently. The aromatic residue Trp79 established 57% hydrophobic interaction. Though during simulation Agnuside did not have continuous interaction with the specificity pocket residues of ALR2 initially, 40% of hydrophobic interactions and 83% of hydrogen bond interactions were established with Leu300 and Leu301, respectively. The hydrogen bonding of Tyr48 was not maintained during the event. The active site residue of Ser302 established 82% of hydrogen bond interactions. Eupalitin-3-O-galactoside retained 30% of hydrophobic interactions with Phe122 of ALR2. Picroside II retained the hydrogen bond interactions with Tyr48, Lys21, and Trp20 with 40%, 18%, and 80% consistency. In addition to this, apolar residues, Leu300 and Phe122, of the active site maintained 45% and 80% consistency, respectively, throughout the simulation period. 7-O-Methylwogonin maintained 11% of hydrogen bond interactions with Leu300.

Sorbinil, a potent ARI, was withdrawn after clinical trials due to lack of specificity resulting from a similar pattern of interaction with ALR2 enzyme and ALR1 with similar IC_50_ values. Interaction of Leu300 of the ALR2 with the inhibitor is the key determinant of specificity of ALR2 over ALR1.

### 3.4. 3-(4,5-Dimethylthiazol-2-yl)-2, 5-diphenyltetrazolium Bromide (MTT) Cell Toxicity Assay

The toxicity of the shortlisted ALR2 inhibitors was assessed using the ARPE-19 cells. The ligands shortlisted for ALR2 inhibition did not show toxicity below 1 *μ*M concentration on ARPE-19 cells. Epalrestat, Agnuside, and 7-O-Methylwogonin did not show toxicity below 5 *μ*M concentrations. The nontoxic nanomolar (nM) concentrations of the compounds were used to test ALR2 inhibition.

### 3.5. Determination of ALR2 Enzyme Activity from Diabetic and Nondiabetic Human Lens Samples

The specific activity of ALR2 was determined from both diabetic and nondiabetic crude human lens samples. The specific activity of ALR2 of the protein sample of diabetic and nondiabetic lens was 0.51 and 0.32 U/mg, respectively.

### 3.6. Inhibition of ALR2 Activity by Identified Phytocompounds in Human Lens Samples

**Figure 1:**
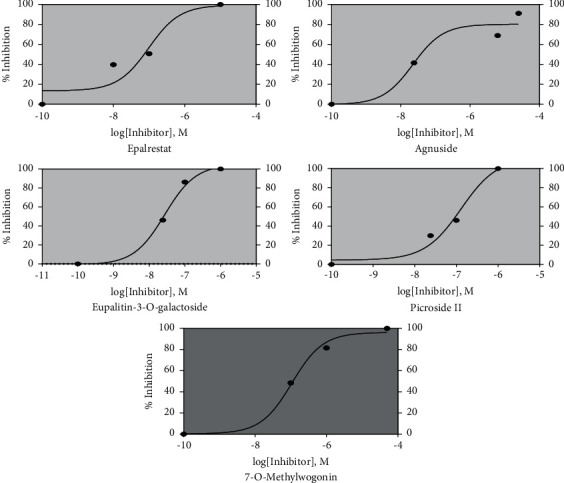
Dose dependent response of Epalrestat and the identified phytocompounds on ALR2 inhibition.

ALR2 activity was assayed spectrophotometrically by measuring the decrease in the absorption of NADPH at 340 nm per unit time at 37°C at pH 7.0.  Figure 1 illustrates the dose response curve of the identified inhibitors of ALR2. The tested compounds inhibited ALR2 in nM concentrations similar to the drug Epalrestat. Recent studies on inhibition of ALR2 using Epalrestat showed 65% of ALR2 inhibition under high glucose condition, proving the potency of Epalrestat in treating DR [43]. Agnuside and Eupalitin-3-O-galactoside inhibited ALR2 protein from diabetic human lens with IC_50_ values lower than that of Epalrestat (98 nM) (Table 6). Agnuside had the lowest IC_50_ of 22.4 nM followed by Eupalitin-3-O-galactoside having an IC_50_ of 27.3 nM in inhibiting human ALR2. 7-O-Methylwogonin had an IC_50_ of 108 nM, and Picroside II had an IC_50_ of 130 nM in inhibiting ALR2.

### 3.7. ALR2 Inhibition in ARPE-19 Cells Using Phytocompounds

**Figure 2:**
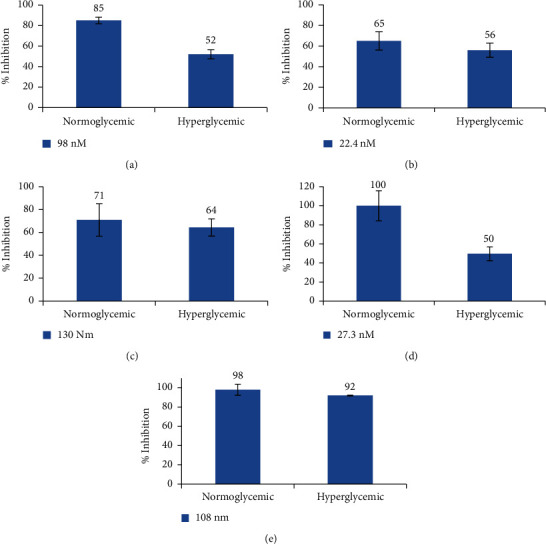
ALR2 inhibition by the identified phytocompounds on normoglycemic and hyperglycemia-induced ARPE-19 cells. (a) Epalrestat, (b) Agnuside, (c) Picroside II, (d) Eupalitin-3-O-galactoside, (e) 7-O-Methylwogonin.

Several studies have been designed for the identification of novel inhibitors of ALR2, including designing derivatives of chemical inhibitors [44]. Compounds that inhibit ALR2 in a pattern similar to Epalrestat are much needed to control complications in DR. Inhibition of ALR2 by the identified phytocompounds in ARPE-19 cells was performed to validate the IC_50_ concentration of the identified compounds on ALR2 from human lens. The inhibition assay was replicated in ARPE-19 cells to ensure the reliability of the result in the human cell system. ALR2 inhibition at the determined IC_50_ concentration of each phytocompound was performed at normoglycemic and hyperglycemic conditions. The inhibition of ALR2 was greater in the cells grown under normoglycemic condition than in the cells grown under hyperglycemic conditions due to the less susceptibility of the hyperglycemia-induced, activated ALR2 protein. The ALR2 in the normoglycemic ARPE-19 cells was inhibited more potentially by Epalrestat, and the phytocompounds Eupalitin-3-O-galactoside, Picroside II, Agnuside, and 7-O-Methylwogonin established 85%, 65%, 71%, 100%, 98% of ALR2 inhibition, respectively. During hyperglycemic conditions, the binding of NADPH to the ALR2 protein changes the conformation of the native enzyme, activating the enzyme and making it less susceptible to the ARIs [45]. The inhibitor must be specific and potent to elucidate ALR2 inhibition. Clinical trials on ARIs indicate that a partial inhibition of ALR2 is sufficient to cause a therapeutic response [46]. All tested compounds exhibited more than 50% inhibition of ALR2 enzyme in ARPE-19 cells, since ARPE-19 cells facilitate entry of nano- and microparticles in a nonsaturable manner [47]. The graphs in Figure 2 represent values obtained from three different experiments, each performed in triplicate. All tests were two-sided with *P* value ≤0.05 indicating the statistical significance of the values. The ALR2 inhibition (%) in hyperglycemic ARPE-19 cells is expressed as means ± SD for *n* = 3.

## 4. Conclusion

The pathological role of ALR2 in diabetes is evident through extensive research for the past 50 years. Though numerous ARIs were employed in the treatment of diabetic complications initially, few came to light as drugs. Identification of specific ARIs has gained importance in research since most of the tested ARIs were withdrawn due to adverse side effects, mainly due to the nonspecific binding of the ARIs to the AKR family of proteins, having a high structural similarity to ALR2. This study employed in silico and in vitro approaches to identify and validate specific and potent ALR2 inhibitors from nature. The ARIs identified through in silico methods showed potency equal to or better than drug Epalrestat, the leading drug used for treating diabetic complications, especially DR. Though Epalrestat has been used worldwide for treating DR, there are side effects of major concern during the long course of usage. The identified compounds, Eupalitin-3-O-galactoside, Picroside II, Agnuside, and 7-O-Methylwogonin, established good inhibition effect on the ALR2 protein of the human lens and ARPE-19 cells. All the identified ARIs had IC_50_ values in the nM range, where Agnuside and Eupalitin-3-O-galactoside had less IC_50_ values (22.4 nM and 27.3 nM, respectively) compared to Epalrestat, indicating the high potency of these compounds in inhibiting human ALR2. Among the four compounds tested for in silico specificity, Agnuside and Picroside II exhibited stable interactions with Leu300, the key residue of ALR2 involved in specific binding, indicating that they might have higher potency towards ALR2 protein than ALR1 and AKR1B10. The inhibition of ALR2 from human lens by the identified compounds was further confirmed using ARPE-19 cells. This work confirms the discovery of novel and specific natural ALR2 inhibitors that offer hope for treating DR in near future.

ALR2 inhibition, though a very old topic of research, has not benefited much from the progress in the identification of novel compounds that can be used as drugs for the control of DR. In silico approaches could pave the way for rapid identification of inhibitors of ALR2 that could pick specific inhibitors, similar to a needle in a haystack, from huge libraries of compounds. Our research could further be extended, for the determination of inhibition of other vital factors of DR that influence worsening of DR, along with ALR2.

## Figures and Tables

**Table 1:** Plants with antidiabetic property used for the identification of novel ALR2 inhibitors.

*Acorus calamus*	*Adhatoda vasica*	*Aegle marmelos*	*Aloe vera*
*Alpinia galanga*	*Andrographis paniculata*	*Aristolochia indica*	*Artemisia annua*
*Artemisia indica*	*Averrhoa bilimbi*	*Azadirachta indica*	*Bacopa monnieri*
*Basella alba*	*Berberis aristata*	*Betula pubescens*	*Boerhavia diffusa*
*Boesenbergia pandurata*	*Boswellia serrata*	*Butea monosperma*	*Capsicum annum*
*Carum carvi*	*Cassia angustifolia*	*Cedrus deodara*	*Crataeva nurvala*
*Curcuma longa*	*Cymbopogon citratus*	*Eclipta alba*	*Embelia ribes*
*Enicostema littorale*	*Eucalyptus globulus*	*Ferula asafetida*	*Ficus religiosa*
*Helicteres isora*	*Lycopersicon esculentum*	*Mucuna pruriens*	*Murraya koenigii*
*Ocimum sanctum*	*Panax ginseng*	*Peganum harmala*	*Phyllanthus amarus*
*Phyllanthus emblica*	*Picrorhiza kurroa*	*Piper nigrum*	*Psoralea corylifolia*
*Pterocarpus marsupium*	*Punica granatum*	*Rauwolfia serpentina*	*Rubia cordifolia*
*Ruta graveolens*	*Saccharum officinarum*	*Stevia rebaudiana*	*Syzygium aromaticum*
*Syzygium cumini*	*Terminalia arjuna*	*Terminalia bellirica*	*Terminalia chebula*
*Trigonella foenum-graecum*	*Vitex negundo*	*Withania somnifera*	

**Table 2:** Compounds shortlisted with higher affinity for ALR2 protein, using Glide docking approach.

Compounds	Extra precision (XP) Glide score (kcal/mol)
Epalrestat	−7.641
Eupalitin-3-O-galactoside	−14.425
Andrographolide	−13.490
Picroside II	−13.221
Agnuside	−12.445
Epicatechin-3-gallate	12.434
7-O-Methylwogonin	−11.736
Negundoside	−11.557
Chlorogenic acid	−11.231
Hexahydrocurcumin	−11.140
*β*-Glucagallin	−11.138
Bisdemethoxycurcumin	−11.117
Luteolin	−10.954
Quercetin dihydrate	−10.841
Galangin	−10.644
Neoandrographolide	−10.501

**Table 3:** Ligand binding free energies recorded for the protein-ligand complexes after IFD of the identified compounds with AKR super family of proteins.

Compound	IFD Glide score (kcal/mol)		
	ALR2	AKR1B10	ALR1
Epalrestat	−9.147	−7.242	−8.516
Eupalitin-3-O-galactoside	−16.266	−7.340	−8.255
Agnuside	−15.302	−11.711	−9.058
Picroside II	−13.000	−10.363	−8.045
7-O-Methylwogonin	−11.968	−10.114	−8.255

**Table 4:** IFD interactions of the identified compounds to determine specificity of the compounds towards ALR2 among AKR super family proteins.

Compound	ALR2	AKR1B10	ALR1
Epalrestat	Tyr48, Trp111, Hie110	Tyr49	Trp114, Arg312
Eupalitin-3-O-galactoside	Tyr48, Hie110, Phe122, Val297, Ala299, Ser302	Tyr49	Trp22, Tyr116, Tyr50
Agnuside	Tyr48, Trp219, Ser302	Tyr49	Trp22, Trp220, Trp114, Ala219
Picroside II	Trp20, Lys21, Val47, Trp48, Hie110, Ala299	Tyr49, Trp21, Cso299	Tyr50, Met302, Arg309
7-O-Methylwogonin	Hie110, Leu300, Leu301	Trp21	Trp114, Met302, Phe125

**Table 5:** Average RMSD and RMSF of the ALR2-phytocompound complexes after 100 ns simulation event.

Protein-ligand complex	Average RMSD (Å)	Average RMSF (Å)
ALR2-Epalrestat	1.95	0.86
ALR2-Agnuside	1.41	0.67
ALR2-Eupalitin-3-O-galactoside	1.45	0.86
ALR2-Picroside II	1.56	0.75
ALR2-7-O-Methylwogonin	1.45	0.74

**Table 6:** The IC_50_ values of the identified inhibitors of ALR2 human lens.

Compound name	IC_50_ (nM)
Epalrestat	98.0 nM ± 5.9
Agnuside	22.4 nM ± 8.0
Picroside II	130.0 nM ± 12.0
Eupalitin-3-O-galactoside	27.3 nM ± 5.13
7-O-Methylwogonin	108.0 nM ± 10.56

The IC_50_ values are expressed as means ± SD for *n* = 3.

## Data Availability

The data used to support the findings of this study are available from the corresponding authors upon request.

## Abbreviations

AGEs: Advanced glycation end products
ALR1: Aldehyde reductase
AKRs: Aldo-ketoreductases
ALR2: Aldose reductase
ARIs: Aldose reductase inhibitors
AKR1B10: Aldose reductase–like protein
DR: Diabetic retinopathy
MD: Molecular dynamics
MTT: 3-(4, 5-Dimethylthiazol-2-yl)-2, 5-diphenyltetrazolium bromide
NADPH: Nicotinamide adenosine dinucleotide phosphate
NF-Κb: Nuclear factor-*κ*appaB
PI3-Akt: Phosphatidylinositol-3-kinase/Akt signaling
ROS: Reactive oxygen species
VEGF: Vascular endothelial growth factor.

## Additional Points

Accession Codes: PDB code for ALR2 complexed with Epalrestat is 4JIR. PDB code for AKR1B10 complexed with Epalrestat is 4JIH. PDB code for ALR1 complexed with Fidarestat is 3H4G.
